# Supplementary figures and images for: Technique and early results of endovenous laser ablation in morphologically complex varicose vein recurrence after small saphenous vein surgery
Source: PLoS One. 2024 Oct 11;19(10):e0310182. doi: 10.1371/journal.pone.0310182 (PMC11469504; doi:10.1371/journal.pone.0310182)

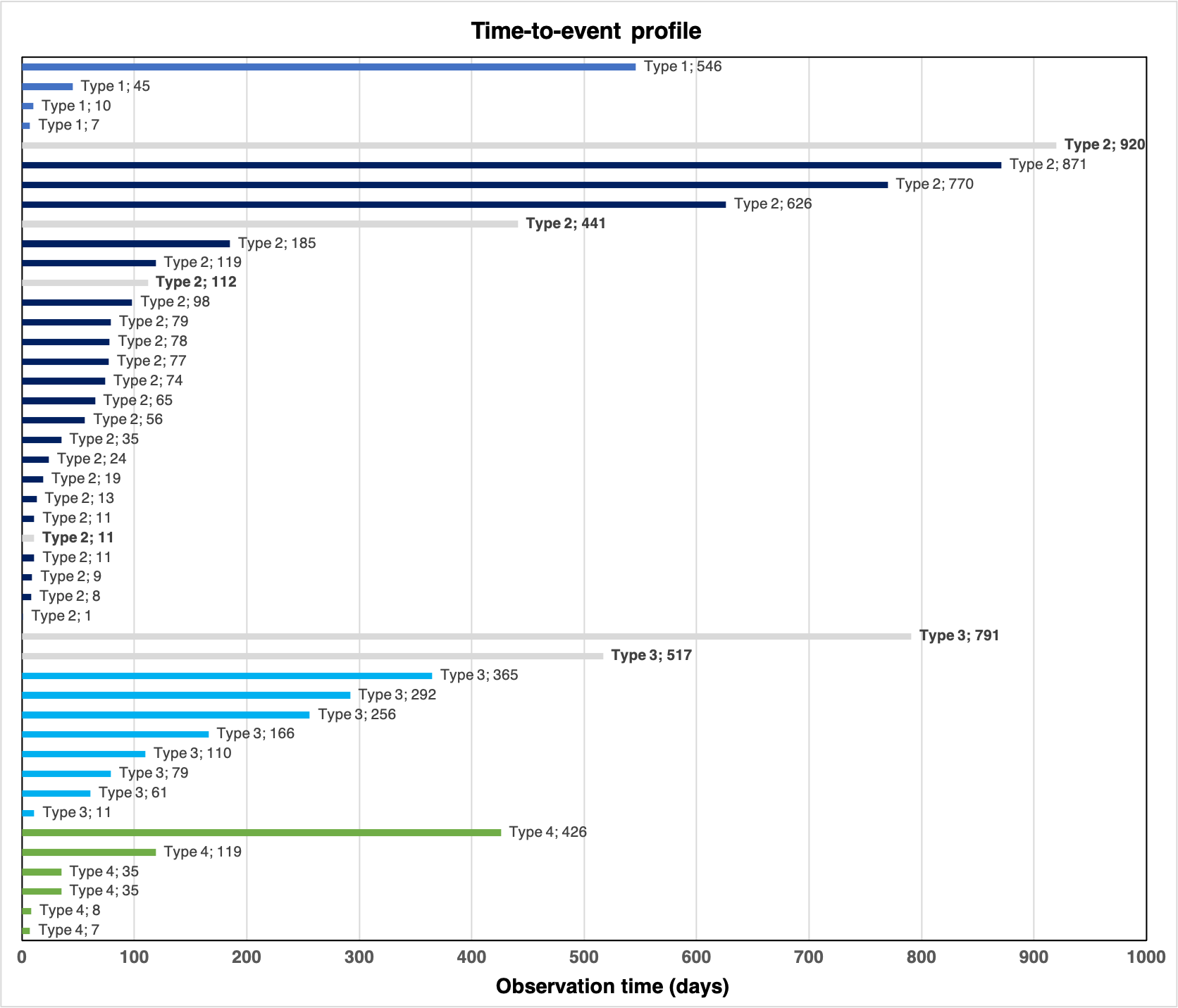

Supplement: S1 Fig — Cases are grouped within the four different morphological types and color-coded. Gray bars are cases with indication for reintervention. The morphologic type and observation time to event or censoring time are indicated next to the bars. (TIF) [file pone.0310182.s001.tif]
