# Supplementary material for: Technique and early results of endovenous laser ablation in morphologically complex varicose vein recurrence after small saphenous vein surgery
Source: PLoS One. 2024 Oct 11;19(10):e0310182. doi: 10.1371/journal.pone.0310182 (PMC11469504; doi:10.1371/journal.pone.0310182)
Supplement: Attachment — Submitted filename: Response_to_Reviewers.docx [file pone.0310182.s003.docx]

***1.PLOS ONE's style requirements, including those for file naming.***

-We have checked and adapted these style requirements

***2. Funding Statement***

- The correct funding statement is:

Parts of our author group (L.M. and S.K.) are employed by Dermatologikum Hamburg GmbH, a non-academic, commercial company. The employer and funder provided support in the form of salaries for these authors [L.M., S.K.], but did not have any additional role in the study design, data collection and analysis, decision to publish, or preparation of the manuscript. The specific roles of these authors are articulated in the ‘author contributions’ section.

***2.Competing Interests Statement***

- Here is the adapted Competing Interests Statement:

Parts of our author group (L.M. and S.K.) are employed by Dermatologikum Hamburg GmbH, a non-academic, commercial company.

L.M. received lecture fees and financial support for travel expenses and congress visits by Biolitec AG

The commercial affiliations displayed do not alter our adherence to PLOS ONE policies on sharing data and materials.

***3. Please upload a new copy of Figures 2, 3, and 4 as the detail is not clear.***

-We have reworked these images using the PACE software and hope that the quality is now OK.

***Review Comments to the Author***

***Reviewer #1: The draft is describing the method for treating a recurrent SSV, but unfortunately , this method is not reproducible and is not cost-effective for majority of patients. However, it merits wide exposure for the specialists in the field.***

- Thank you for your comment. We agree that reproducibility and cost-effectiveness is something that was not mentioned in the previous manuscript. We have made further comments on this in the Limitations section. We have also mentioned the need for clinical efficacy and cost-effectiveness in the Conclusions.

***Reviewer #2:***

***Title: OK***

***Abstract. Background: Please briefly describe the clinical setting and current status of venous disease. This is not a specialized journal and this could help readers***

- The abstract was expanded accordingly at the beginning under Background.

***Methods: Remove case series, just leave retrospective study***

***Remove N***

***Add briefly your statistical tests in this section.***

***Spell out nm when using for the fist time in your abstract***

***Remove many and leave the absolute or exact number (35)***

-Thank you for these suggestions for changes to the abstract, which we have tried to implement as best as possible.

***Introduction.***

***Overall well-written, although I suggest to situate more in context in regards venous disease, stages and CEAP.***

-We have included more background on superficial venous insufficiency at the beginning of the introduction. In addition, two further literature references have been added to the manuscript.

***Methods: There is no need to leave Case series, please remove.***

-We have removed this.

***Figure 1, label A-D, indicates SPJ and vessel. Were all procedures bilateral?***

-We have adapted Figure 1 accordingly. We have also added additional labels for the structures and the vessel direction (prox-dist). The caption for Figure 1 has also been expanded accordingly.

***Figure 3. From A to D is hard to visualize. Perhaps separating the E could help.***

-We hope that by re-uploading the image using PACE we will achieve improved quality here.

***Figure 5, use arrows.***

-We did not understand this comment exactly. We think the necessary information is included in the Kaplan-Meier curve or displayed below the curve. We do not know where the arrows should be placed here.

***Results: Very well presented and described.***

***Discussion: This is often regarded as the most important section of the manuscript, where authors complete a deep analysis of their findings, compare with previous reports, point out learning strategies and propose further direction.***

***Overall well written.***

-Thank you for your evaluation.

***Reviewer #3:***

***This study evaluates the technical feasibility and early outcomes of endovenous laser ablation (EVLA) for complex varicose vein recurrences post-small saphenous vein surgery. The retrospective case series of 45 EVLA procedures showed a 97.8% initial success rate, with 13.3% requiring re-intervention during a median 77-day follow-up. The study concludes that EVLA is feasible for these complex cases, albeit with a lower success rate than primary treatments, and without significant complications. But there are still some limitations:***

***1. As this is a single-center retrospective study, its findings may be influenced by selection bias and the experience of a single surgeon. The study included only 45 EVLA procedures, a relatively small sample size that might affect the power of the statistical results and their generalizability.***

-We agree with this assessment and have explained this point under Limitations in the revision. Even if the statistical power is limited by a small number of cases, we currently have no better data in the literature. The data situation would improve if practitioners were to publish their undoubted experience.

***2. The median follow-up time of 77 days might not be sufficient to assess the long-term effects and potential long-term complications of EVLA. Some patients did not attend further examinations after the first follow-up, which could lead to incomplete follow-up data, affecting the accuracy of the results.***

-We pointed out, also under Limitations, that no conclusions on long-term results can be drawn from the data:

*The median follow-up period of 77 days is inherently insufficient to assess the long-term effects and potential long-term complications of EVLA. Several patients did not participate in further examinations after the first follow-up. However, complete follow-up outside of prospective studies is not easy to realize as part of the routine treatment of outpatients with varicose veins. Retrospective data can nevertheless be useful for planning prospective studies. It must also be mentioned that the report represents only the experience of a single surgeon from a single center. This can influence the results through biased selection. On the other hand, this setting results in a high degree of standardization and potentially a low information bias.*

***3. I would suggest conducting a multifactorial analysis to assess other variables that may affect the results, such as patient characteristics, lifestyle, and comorbidities.***

- For subgroup analyses, even with regard to univariate analyses, the number of cases and observations appeared too low. We therefore deliberately left all cases in one cohort.

The number of cases and observations also appeared too small for possible multivariate analyses, such as Cox proportional hazard or multifactor analysis.

We have also commented on this under Limitations:

*In addition to the small number of cases, the frequency of only six events of re-recurrence during follow-up does not provide a sufficient basis for subgroup analyses, let alone multivariate survival analyses.*

***4. A more in-depth discussion in the discussion section may enhance the manuscript, including how the results compare with existing literature, as well as potential limitations and future research directions.***

-Thank you for the suggestion. As a result, we have significantly expanded the entire paragraph on Limitations, also with regard to the previous comments.

In the discussion, a further technical aspect and a corresponding reference (Ref. 24) were added.

Overall, very few comparable studies or cases have been published to date, as far as is known. To the best of our knowledge, these have been taken into account in the Introduction and Discussion.

The following comments on possible further studies has also been added in the Limitations section:

*Further studies investigating comparability and cost-effectiveness are necessary. To this end, it would be essential to validate methods that quantify reflux or the overall severity of recurrence, for example using duplex sonographic or radiological imaging techniques. This could serve as a basis for a comparison of the different approaches, e.g. foam sclerotherapy and thermal ablation, in prospective studies. Possibly a threshold value, if such a value exists, can be determined for the extent of the findings above which EVLA is superior to foam sclerotherapy. Another option for evaluating data from routine treatment would be to analyze data from registry studies. However, a general consensus regarding the characterization and quantification of recurrences would also be useful here.*
