# Supplementary material for: Technique and early results of endovenous laser ablation in morphologically complex varicose vein recurrence after small saphenous vein surgery
Source: PLoS One. 2024 Oct 11;19(10):e0310182. doi: 10.1371/journal.pone.0310182 (PMC11469504; doi:10.1371/journal.pone.0310182)
Supplement: Attachment — Submitted filename: Response_to_Reviewers.docx [file pone.0310182.s004.docx]

Dear Dr. Ranjan,

In the following, we would like to address the individual comments made by you and the reviewers.

We have prefaced our comments with each of your comments or points for consideration in ***bold italics.***

***Journal Requirements:***

***Please review your reference list to ensure that it is complete and correct. If you have cited papers that have been retracted, please include the rationale for doing so in the manuscript text, or remove these references and replace them with relevant current references. Any changes to the reference list should be mentioned in the rebuttal letter that accompanies your revised manuscript. If you need to cite a retracted article, indicate the article’s retracted status in the References list and also include a citation and full reference for the retraction notice.***

-We have looked through all the cited publications and can see that none of the papers have been withdrawn as of today's access date (August 12, 2024). However, we have replaced reference 14 with another article.

***Review Comments to the Author:***

***Reviewer #2: Authors have addressed well my initial concerns.***

***Minor observation and important. Please add in you abstract, the number of patients, mean age, SD and gender proportions.***

- We have included this information in the abstract.
